# Cepharanthine Reduces Endometriosis Lesions and Alters Mitochondrial and Autophagy-Related Signals in Endometriotic Stromal Cells

**DOI:** 10.3390/molecules31152722

**Published:** 2026-08-05

**Authors:** Miji Kim, Wonhyoung Park, Hee Seung Kim, Whasun Lim, Gwonhwa Song, Sunwoo Park

**Affiliations:** 1 Agri-Food Bio Convergence Institute, Gyeongsang National University, Jinju 52725, Republic of Korea; mj-kim@korea.ac.kr; 2 Department of Animal Science, Chungbuk National University, Cheongju 28644, Republic of Korea; whpark@chungbuk.ac.kr; 3 Department of Obstetrics and Gynecology, Seoul National University Hospital, Seoul 03080, Republic of Korea; bboddi0311@snu.ac.kr; 4 Department of Obstetrics and Gynecology, Seoul National University College of Medicine, Seoul 03080, Republic of Korea; 5 Department of Biological Sciences, College of Science, Sungkyunkwan University, Suwon 16419, Republic of Korea; wlim@skku.edu; 6 Department of Biotechnology, College of Life Sciences and Biotechnology, Korea University, Seoul 02841, Republic of Korea; 7 Department of GreenBio Science (BK21), Gyeongsang National University, Jinju 52725, Republic of Korea

**Keywords:** endometriosis, cepharanthine, autophagy, mitochondrial function, oxidative stress

## Abstract

Endometriosis is a chronic inflammatory disorder characterized by the ectopic growth of endometrial-like tissue, leading to pelvic pain and infertility. Although cepharanthine has well-established anti-inflammatory properties, its therapeutic potential and underlying mechanisms in endometriosis remain largely unexplored. In this study, the effects of cepharanthine were evaluated using a surgically induced mouse model and immortalized human endometrial stromal cell models. Cepharanthine treatment significantly reduced the calculated lesion volume, whereas wet lesion weight did not differ significantly between the groups. Cepharanthine was also accompanied by reduced spleen weight and alterations in the CD4+ helper T-cell population in vivo. In immortalized human ovarian endometriotic stromal cells (ihOESCs), cepharanthine significantly decreased cell viability, induced apoptosis, and disrupted cell-cycle progression. Cepharanthine altered autophagy-related signaling, accompanied by increased acidic vesicle-associated signals and accumulation of LC3B-II and p62/SQSTM1; however, the direction of autophagic flux remains unclear. These changes were associated with elevated reactive oxygen species production, intracellular Ca^2+^ redistribution, and mitochondrial dysfunction. Collectively, these findings indicate that cepharanthine reduces calculated lesion volume in vivo and alters mitochondrial function, autophagy-related signaling, apoptosis and cell-cycle progression in ihOESCs, supporting its potential as a therapeutic candidate.

## 1. Introduction

Endometriosis is a disease in which endometrial tissue is abnormally present in areas outside the uterus, such as the abdominal cavity or ovaries, and causes an inflammatory response [1]. Although the mechanism underlying the development of endometriosis has not been fully identified, it is often explained by Sampson’s theory of retrograde menstruation [2]. According to this theory, menstrual blood flows backward into the abdominal cavity through the fallopian tubes, and the endometrial cells in the menstrual blood attach and proliferate within the abdominal cavity, forming lesions. Endometriosis treatment primarily involves hormone therapy, surgical removal, or a combination of both; however, conventional methods carry a high likelihood of recurrence and side effects [3]. Therefore, studies are underway to develop noninvasive and non-hormonal treatments.

Cepharanthine is a natural isoquinoline alkaloid known for its anti-inflammatory and antitumor effects in various diseases [4,5]. For example, it can alleviate symptoms of inflammatory conditions, such as LPS-induced systemic inflammation or dextran sulfate sodium (DSS)-induced colitis [4,6]. Cepharanthine can also induce apoptosis by generating reactive oxygen species (ROS) in various tumor cells, including lung cancer, gastric cancer, and myeloma [7,8,9]. In addition, previous studies have suggested that cepharanthine may promote autophagy in various abnormal cells. Cepharanthine at concentrations of 0–10 μM promotes autophagy, cell cycle arrest, and apoptosis by inhibiting the mTOR signaling pathway in breast cancer cell lines [10]. Another study found that cepharanthine may counteract senescence induced by the broad-spectrum anticancer drug doxorubicin in mouse fibroblast cells by activating autophagy [11]. These studies suggest that cepharanthine can activate autophagy-related factor expression and induce apoptosis or cell cycle arrest.

Endometriosis lesions are characterized by dysregulated chronic inflammation, cell proliferation, and tissue remodeling, accompanied by reduced autophagy signaling [12]. The reduction in autophagy, combined with decreased apoptosis signaling and dysregulated immune response, may accelerate the progression of endometriosis [12,13,14]. Autophagy plays a critical role in regulating intracellular metabolic homeostasis, removing damaged organelles, and facilitating the survival or elimination of lesion cells [15]. The autophagy-regulated effects of cepharanthine suggest its potential to improve the pathological environment of endometriosis. This is a key reason we selected cepharanthine as a candidate for the treatment of endometriosis in this study.

In the present study, we evaluated the effects and mechanisms of cepharanthine using a surgically induced endometriosis mouse model as an in vivo model. The mouse model, established using Pelch’s procedure, is widely utilized to study endometriosis and develop appropriate therapeutic strategies [16]. This model reflects the pathophysiology of human disease, including reduced reproductive capacity, impaired fertility, and changes in gene and protein expression. Immortalized human ovarian endometriotic stromal cell lines (ihOESCs) have been used as an in vitro model of endometriosis. This cell line was established by immortalizing primary cells obtained from endometriosis lesions and can replicate the cellular responses and molecular characteristics of these lesions [17]. To address whether cepharanthine affects non-endometriotic stromal cells, we identified the effects of cepharanthine on hTERT-immortalized human endometrial stromal cells (T-HESCs) as a non-endometriotic stromal comparator at the experimental concentration. Together, these in vivo and in vitro models provide valuable tools for evaluating the therapeutic potential of candidate active substances.

The aim of this study was to evaluate the effect of cepharanthine on endometriosis-related endpoints and characterize the cellular stress response induced by cepharanthine in immortalized ectopic stromal cells. First, in vivo experiments were conducted to determine whether cepharanthine treatment could regulate lesion growth and modulate inflammatory and immune responses. Next, in vitro experiments were performed to assess cellular responses, including oxidative stress, mitochondrial function, autophagy-related signaling, and apoptosis induction, to explore the potential of cepharanthine as a candidate for endometriosis.

## 2. Results

### 2.1. Cepharanthine Reduces Calculated Lesion Volume and Alters Autophagy-Related Gene Expression in Mice with Endometriosis

**Figure 1:**
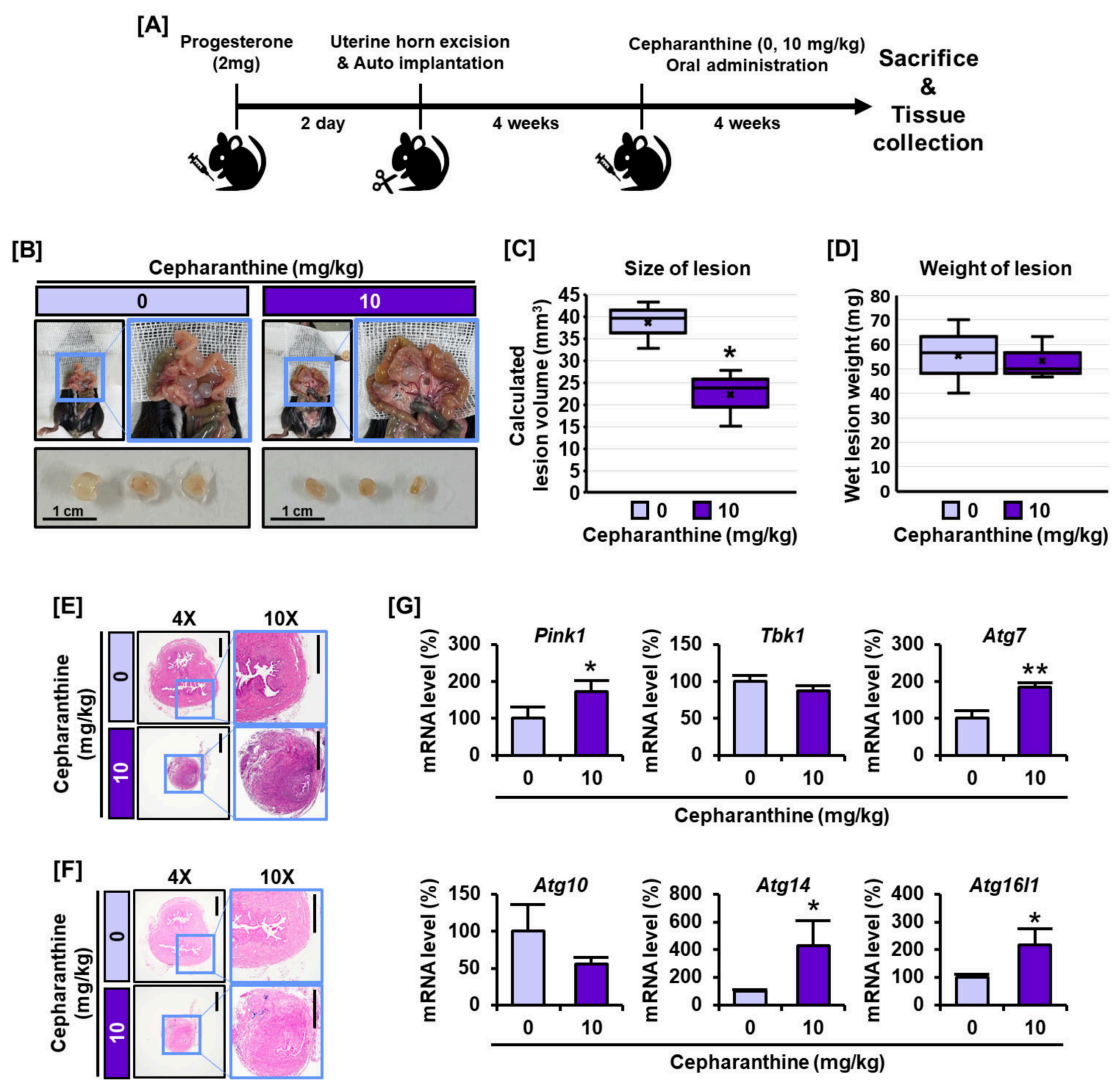
Cepharanthine suppresses lesion growth and modulates autophagy-related responses in the endometriosis mouse model. (**A**) Establishment of an endometriosis mouse model and treatment with cepharanthine are shown in the scheme. (**B**) Endometriosis lesions were collected after the mouse model completed oral administration for 4 weeks with 0 or 10 mg/kg cepharanthine. (**C**) Lesion volume was calculated from the measured length and width using V = (L × W^2^)/2. The mean calculated volume of the three lesions from each mouse was used as one biological replicate. (**D**) Wet endometriosis lesion weight was measured and presented as box and whisker plots. For each mouse, the mean of three lesions was used as one biological replicate. (**E**) The histological analysis of the endometriosis lesion was conducted through hematoxylin and eosin (H&E) staining. (**F**) The iron accumulation in 10 mg/kg cepharanthine-treated lesions was assessed by iron staining. The scale bar represents 500 μm. (**G**) The mRNA expression of the autophagy-related gene was analyzed by quantitative PCR (qPCR). The bar graph presented overexpression of Pink1, Atg7, Atg14, and Atg16l1. All experiments were analyzed with *n* = 3 mice per group. Statistics were analyzed using a two-tailed unpaired Student’s *t*-test. The asterisks (*) indicated statistical significance: * *p* < 0.05 and ** *p* < 0.01.

To evaluate the effectiveness of oral cepharanthine in treating endometriosis, an endometriosis model was established using C57BL/6J mice. The mice were divided into two groups and administered either 0 or 10 mg/kg of cepharanthine orally for 4 weeks (Figure 1A). After the treatment period, the mice were euthanized, and endometriosis lesions were examined. Lesion length (L) and width (W) were measured, and lesion volume (V) was geometrically calculated using V = (L × W^2^)/2. The mean calculated lesion volume was significantly lower in the 10 mg/kg cepharanthine-treated groups than in the 0 mg/kg cepharanthine (0.5% carboxymethylcellulose; CMC)-treated group (Figure 1B). The calculated lesion volume in the 0 mg/kg cepharanthine-treated group was 38.60 mm^3^, whereas it was significantly reduced to 22.24 mm^3^ in the 10 mg/kg cepharanthine-treated group (*t*_4_ = 3.36, *p* = 0.03016) (Figure 1C). Interestingly, no significant difference in wet lesion weight was observed between the two groups (Figure 1D). Histological changes were then examined using H&E staining. In the 0 mg/kg cepharanthine-treated group, lesion growth progressed, forming a cavity within the lesion. In contrast, lesion growth was suppressed in the 10 mg/kg cepharanthine-treated group (Figure 1E). In addition, iron staining analysis revealed iron accumulation within the endometriosis lesions of mice treated with 10 mg/kg cepharanthine (Figure 1F). We investigated the expression of autophagy-related genes within the endometriosis lesions. The mRNA expression levels of *Pink1*, *Atg7*, *Atg14*, and *Atg16l1* increased by 172.46% (*t*_4_ = 3.15, *p* = 0.0346), 184.11% (*t*_4_ = 6.59, *p* = 0.0049), 428.36% (*t*_4_ = 3.29, *p* = 0.0375), and 217.08% (*t*_4_ = 3.73, *p* = 0.0203), respectively (Figure 1G). *Atg10* showed a decreasing trend in lesion tissues; however, this change did not reach statistical significance. These results suggest that cepharanthine is associated with reduced lesion volume and increased expression of autophagy-related genes in lesion tissues.

### 2.2. Cepharanthine Alters the Spleen Weight and Splenic CD4+ T-Cell Proportion in Mice with Endometriosis

**Figure 2:**
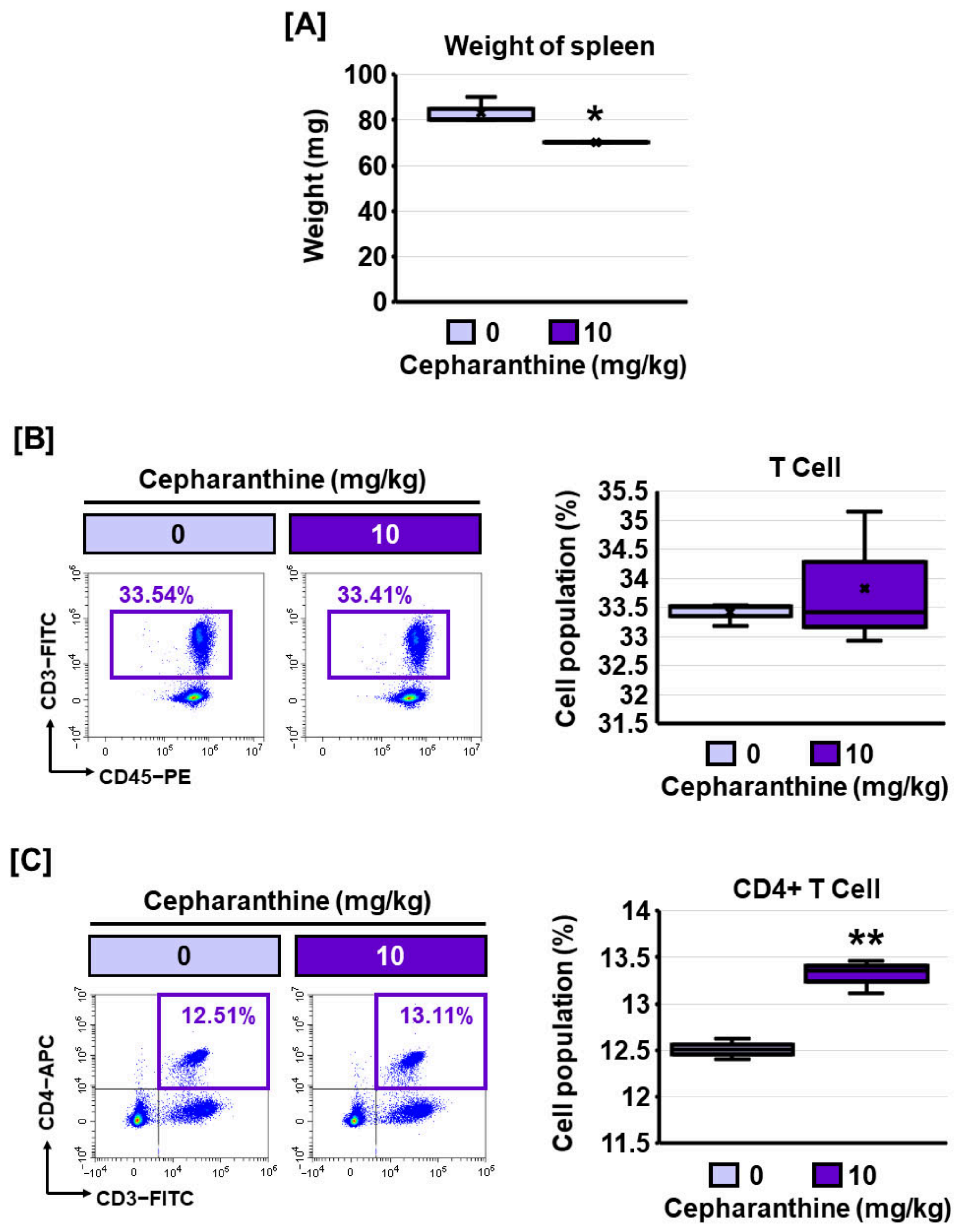
Effects of cepharanthine on spleen weight and splenic T-cell proportions in the endometriosis mouse model. (**A**) The weight of the spleen was measured and presented as a box and whisker plot. (**B**) CD45-PE versus CD3-FITC positive cell population represents the total T cell population. (**C**) CD3-FITC versus CD4-APC positive cell population represents CD4+ helper T cell population. All experiments were analyzed with *n* = 3 mice per group. Statistics were analyzed using a two-tailed unpaired Student’s *t*-test. The asterisks (*) indicated statistical significance: * *p* < 0.05 and ** *p* < 0.01.

Spleen weight and splenic T cell proportions were evaluated as immune-related endpoints in endometriosis-induced mouse models. The spleen weight in the vehicle-treated group was 83.33 mg, and it was reduced to 70.00 mg (*t*_4_ = 16.00, *p* = 0.0286) in the cepharanthine-treated group (Figure 2A). The T cell population was assessed using the lymphocyte marker CD45, the total T cell marker CD3, and the helper T cell marker CD4. The total T cell population was analyzed using CD45 and CD3, and no significant difference was observed between the two groups (Figure 2B). The CD3+CD4+ helper T cell population was significantly altered (*t*_4_ = 6.56, *p* = 0.0053) in the 10 mg/kg cepharanthine-treated group (Figure 2C). Although statistically significant (*p* < 0.01), the increase in CD4+ T-cell population (12.51% to 13.24%) is modest and should be interpreted cautiously given the limited number of biological replicates (*n* = 3). These results indicate treatment-associated change in the measured splenic endpoints.

### 2.3. Cepharanthine Suppresses Cell Viability and Increases Annexin V/PI-Defined Apoptotic Populations in ihOESCs

**Figure 3:**
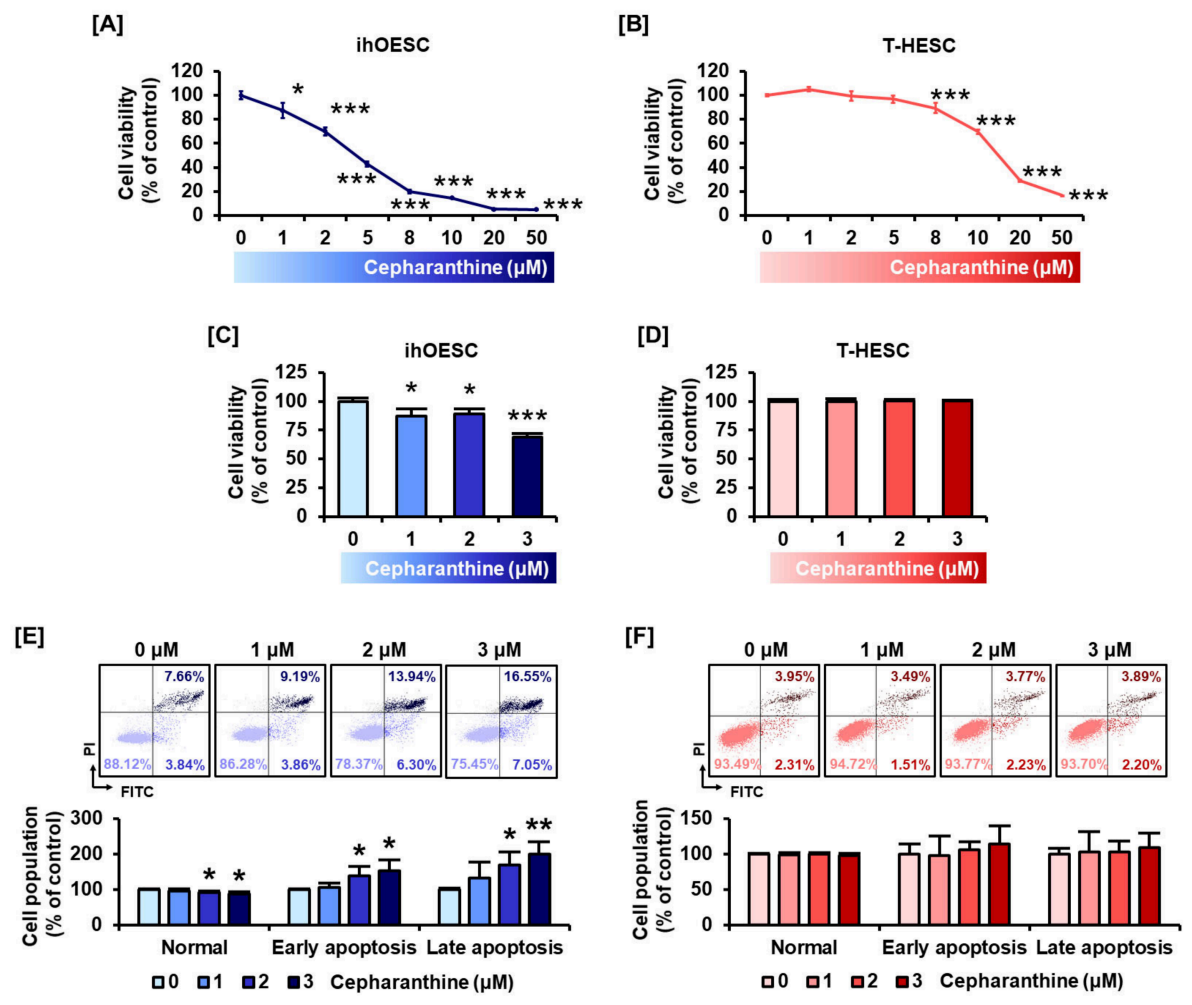
Cepharanthine reduces cell viability and increases apoptotic populations in ihOESCs. (**A**,**B**) MTT-based viability analysis of (**A**) ihOESCs and (**B**) T-HESCs after cepharanthine exposure was performed. (**C**) The viability of ihOESCs was reduced in a concentration-dependent manner. (**D**) The viability of T-HESCs showed no significant changes after cepharanthine treatment. (**E**,**F**) Annexin V/PI apoptosis analysis in (**E**) ihOESCs and (**F**) T-HESCs was performed to identify apoptosis induction after cepharanthine exposure. Statistics were performed by R using one-way ANOVA and Tukey’s test. All experiments were analyzed with biological triplicate. The asterisks (*) indicated statistical significance: * *p* < 0.05, ** *p* < 0.01, *** *p* < 0.001. Abbreviations: ihOESCs, immortalized human ovarian endometriotic stromal cells; T-HESCs, telomerase-immortalized human endometrial stromal cells; MTT, 3-(4,5-dimethylthiazol-2-yl)-2,5-diphenyltetrazolium bromide; PI, propidium iodide.

Cepharanthine can inhibit the growth of endometriosis lesions and regulate the splenic T-cell proportion in endometriosis mouse models. Therefore, we used ihOESCs to examine how cepharanthine affects endometriosis at the cellular level. In parallel, T-HESCs were also analyzed as a non-endometriotic immortalized stromal comparator under the same in vitro conditions. Cells were treated with various concentrations of cepharanthine, and cell viability decreased in a dose-dependent manner (Figure 3A). Specifically, the survival rate decreased to 70.00% of the control (*F*_1,4_ = 116.45, *p* = 0.0004) when treated at 2 μM and to 42.82% of the control (*F*_1,4_ = 586.17, *p* < 0.0001) at 5 μM in ihOESCs. The IC50 value was calculated to be 3.33 μM. Thus, the subsequent experiment was conducted at 3 μM as the optimal concentration. In contrast, T-HESC viability presented no significant changes up to 8 μM under the same conditions (Figure 3B). To confirm that the MTT-based viability outcomes were not due to mitochondrial dysfunction, trypan blue staining was further performed at experimental concentrations (0, 1, 2, and 3 μM). ihOESCs exhibited 69.17% (*F*_1,4_ = 328.531; *p* < 0.0001) relative to the control at 3 μM (Figure 3C). However, T-HESCs presented no significant changes (Figure 3D).

In addition, the decrease in cell viability following cepharanthine treatment may be associated with apoptotic cell death. Therefore, flow cytometry analysis was performed using annexin V/PI staining. The proportion of normal cells decreased to 88.75% of control (*F*_1,4_ =18.97, *p* = 0.0121), while the proportions of early apoptotic and late apoptotic cells increased to 154.67% of control (*F*_1,4_ = 12.19, *p* = 0.0251) and 200.52% of control (*F*_1,4_ = 23.62, *p* = 0.0083) respectively, in ihOESCs treated with 3 μM cepharanthine (Figure 3E). However, T-HESCs treated with 0–3 μM of cepharanthine showed no significant changes in annexin V/PI-stained apoptotic cell populations (Figure 3F).

### 2.4. Cepharanthine Alters Autophagy-Related Signals and Cell-Cycle Distribution in ihOESCs

**Figure 4:**
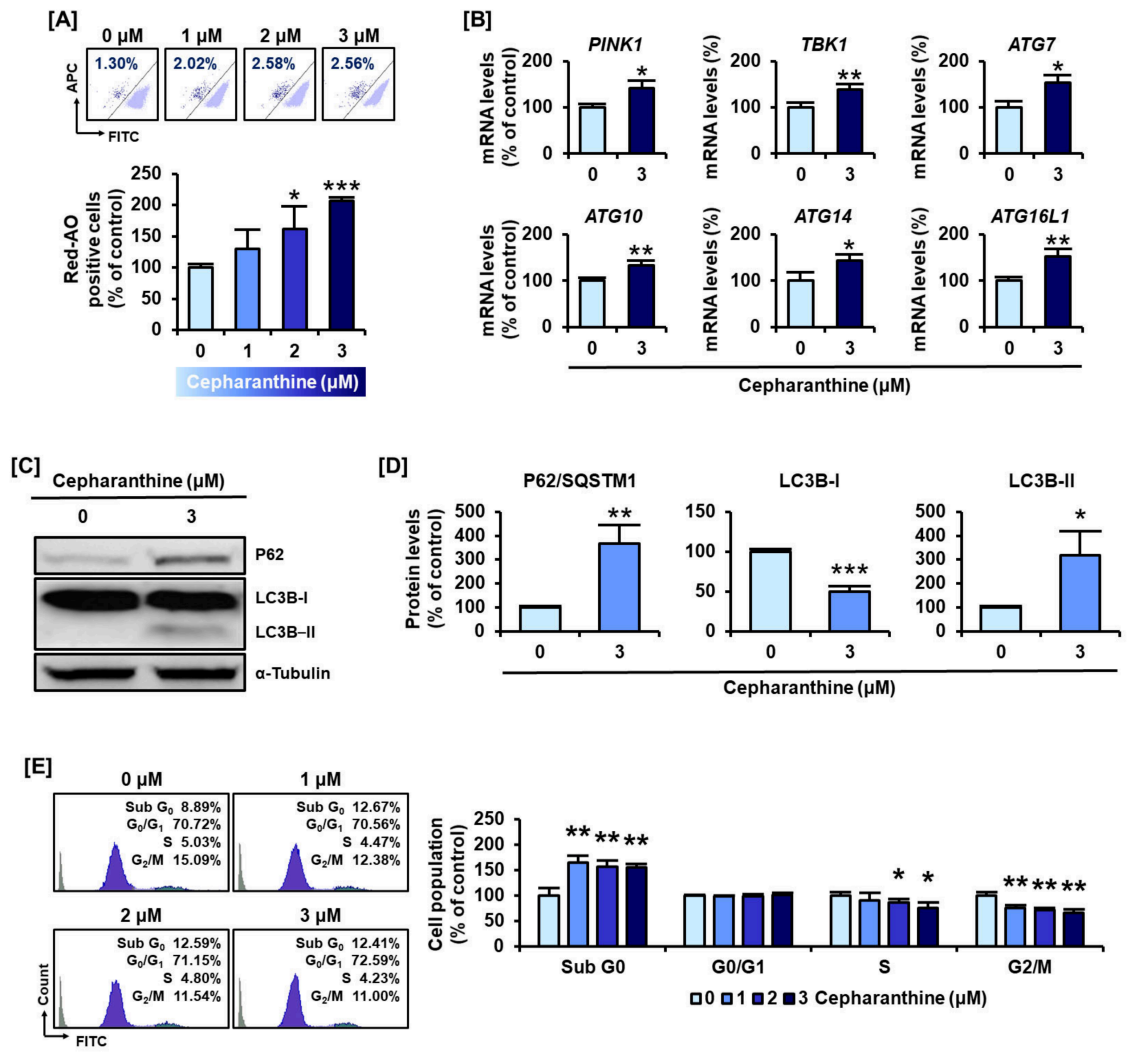
Cepharanthine promotes autophagy-associated changes and alters cell-cycle distribution in ihOESCs. (**A**) Acridine orange (AO) staining and quantification of red-AO-positive cells were conducted to identify acidic vesicle-associated signals in ihOESCs. (**B**) The mRNA expression levels of autophagy-related factors (PINK1, TBK1, ATG7, ATG10, ATG14, and ATG16L1) were analyzed. (**C**) The protein levels of LC3B-I and LC3B-II were assessed through Western blotting analysis. (**D**) The LC3B-II/LC3B-I ratio was calculated and presented as a bar graph. (**E**) The cell cycle distribution was analyzed by PI staining in cepharanthine-exposed ihOESCs. Three independent biological experiments were conducted. Statistical analyses between three or more groups were performed using the R program 4.3.0. using one-way ANOVA and Tukey’s test. Statistics between two groups were analyzed using a two-tailed unpaired Student’s *t*-test. The asterisks (*) indicated statistical significance: * *p* < 0.05, ** *p* < 0.01, *** *p* < 0.001.

Acridine orange (AO) staining was performed to determine whether cepharanthine alters acidic vesicle-associated signals in ihOESCs. The relative proportion of red AO-positive cells increased to 161.62% of the control (*F*_1,4_ = 8.98, *p* = 0.0401) at 2 μM and 206.27% of the control (*F*_1,4_ = 498.94, *p* < 0.0001) at 3 μM cepharanthine treatment (Figure 4A). The increase in AO-positive cells suggests altered acidic vesicle-associated signals after cepharanthine exposure. The mRNA expression levels of the autophagy-related genes were also assessed (Figure 4B). The expression of *PINK1*, *TBK1*, *ATG7*, *ATG10*, *ATG14*, and *ATG16L1* was significantly increased after cepharanthine treatment.

The protein levels of p62/SQSTM1, LC3B-I, and LC3B-II were further evaluated with Western blotting (Figure 4C). Cepharanthine treatment increased p62/SQSTM1 expression to 366.26% (*F*_1,4_ = 36.26, *p* = 0.0038) and LC3B-II expression to 319.45% (*F*_1,4_ = 14.77, *p* = 0.0184) of the control, whereas LC3B-I expression decreased to 49.38% (*F*_1,4_ = 118.68, *p* = 0.0004). These results suggest that cepharanthine treatment alters autophagy-related signaling. Because a complete dynamic flux assay was not conducted, the direction of autophagic flux remains unresolved. However, accumulation of LC3B-II and p62/SQSTM1 may reflect reduced autophagic degradation.

In addition, since the decrease in cell viability could also be related to proliferation inhibition, the cell cycle distribution was analyzed (Figure 4D). The G0/G1 distribution remained unchanged following cepharanthine treatment. However, in the 3 μM cepharanthine-treated group, the S phase decreased to 75.16% of control (*F*_1,4_ = 14.34, *p* = 0.0193), the G2/M phase decreased to 65.52% of control (*F*_1,4_ = 40.98, *p* = 0.0031), and the SubG0 phase, indicative of DNA fragmentation, increased to 155.05% of control (*F*_1,4_ = 32.96, *p* = 0.0046). These results indicate that cepharanthine alters cell-cycle distribution in ihOESCs.

### 2.5. Cepharanthine Disrupts Mitochondrial Function in ihOESCs

**Figure 5:**
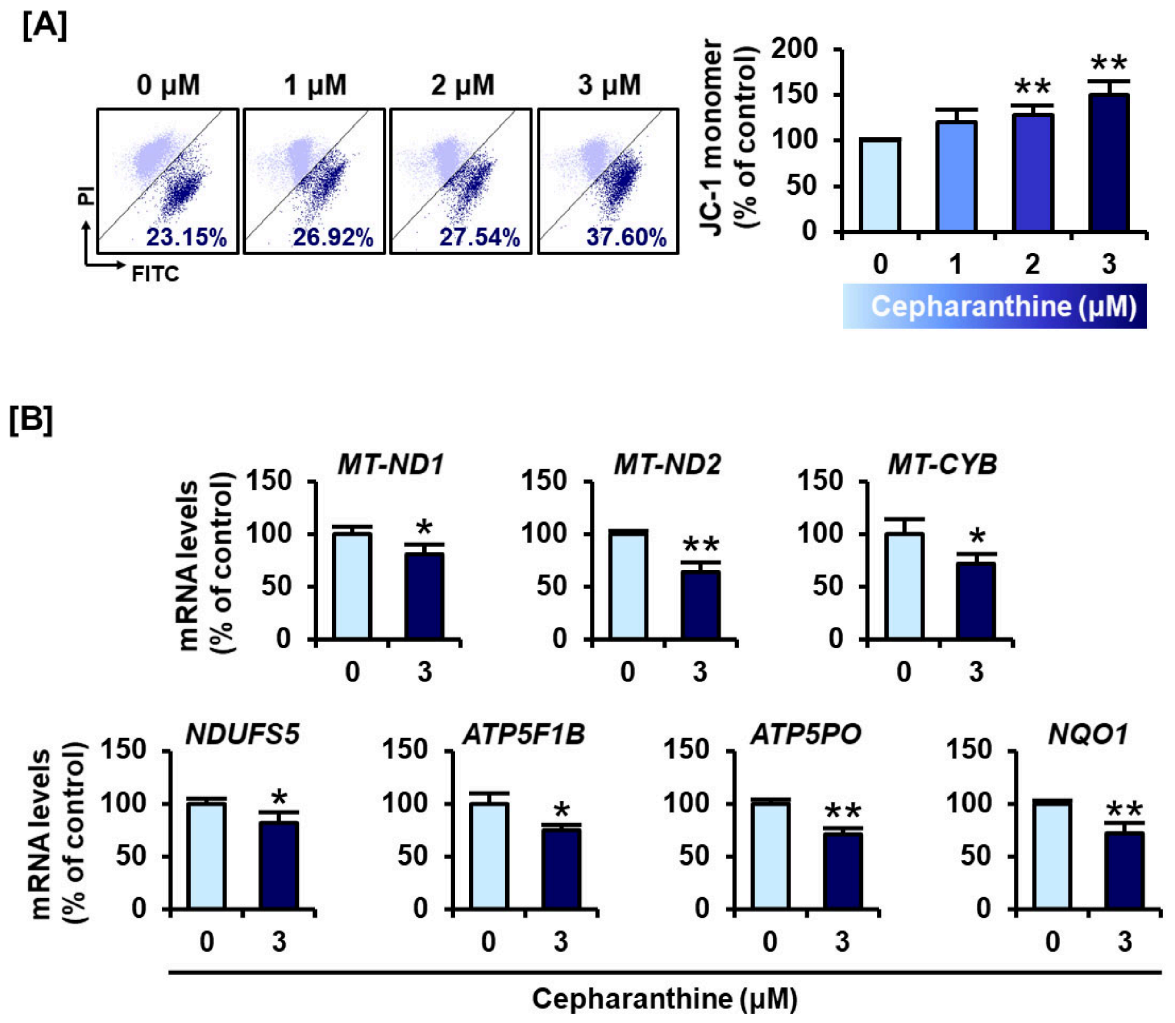
Cepharanthine impairs mitochondrial function in ihOESCs. (**A**) The mitochondrial membrane potential (MMP) was identified by JC-1 staining. The relative levels of JC-1 monomer, representing the MMP depolarization, were increased after cepharanthine treatment. (**B**) The mRNA expression of mitochondrial function-related factors (MT-ND1, MT-ND2, MT-CYB, NDUFS5, ATP5F1B, ATP5PO, and NQO1) was downregulated in cepharanthine-treated ihOESCs. Three independent biological experiments were conducted. Statistical analyses between three or more groups were performed with R programs using one-way ANOVA and Tukey’s test, comparing all pairs of group averages. The asterisks (*) indicated statistical significance: * *p* < 0.05 and ** *p* < 0.01.

Mitochondria are key intracellular organelles involved in the induction of apoptosis and autophagy. Therefore, we assessed the MMP to evaluate mitochondrial function. The ratios of JC-1 monomers increased to 127.57% of control (*F*_1,4_ = 23.67, *p* = 0.0082) and 149.65% (*F*_1,4_ = 38.33, *p* = 0.0035) after treatment with 2 μM and 3 μM cepharanthine, respectively (Figure 5A). An increase in JC-1 monomers indicates depolarization, reflecting impaired mitochondrial function. MMP is linked to the functions of the electron transport chain (ETC) and ATP synthase. Consequently, we analyzed the expression of the mitochondrial function-related genes (Figure 5B). The ETC-related genes *MT-ND1*, *MT-ND2*, *MT-CYB*, and *NDUFS5* were expressed at 80.72% (*t*_4_ = 3.42, *p* = 0.0268), 63.97% (*t*_4_ = 7.23, *p* = 0.0019), 71.59% (*t*_4_ = 3.28, *p* = 0.0306) and 81.72% (*t*_4_ = 3.53, *p* = 0.0242), respectively, in cepharanthine-treated ihOESCs. In addition, *ATP5F1B* was reduced to 74.60% (*t*_4_ = 3.92, *p* = 0.0172), and ATP5PO was reduced to 71.15% (*t*_4_ = 32.86, *p* = 0.0046). Finally, the expression of *NQO1*, a gene involved in mitochondrial redox balance, was also reduced to 71.60% (*t*_4_= 6.44, *p* = 0.0030) following cepharanthine treatment. These results suggest mitochondrial membrane depolarization with reduced expression of mitochondrial function-related genes in ihOESCs.

### 2.6. Cepharanthine Increases Oxidant-Sensitive Fluorescence and Alters Ca^2+^ Distribution in ihOESCs

**Figure 6:**
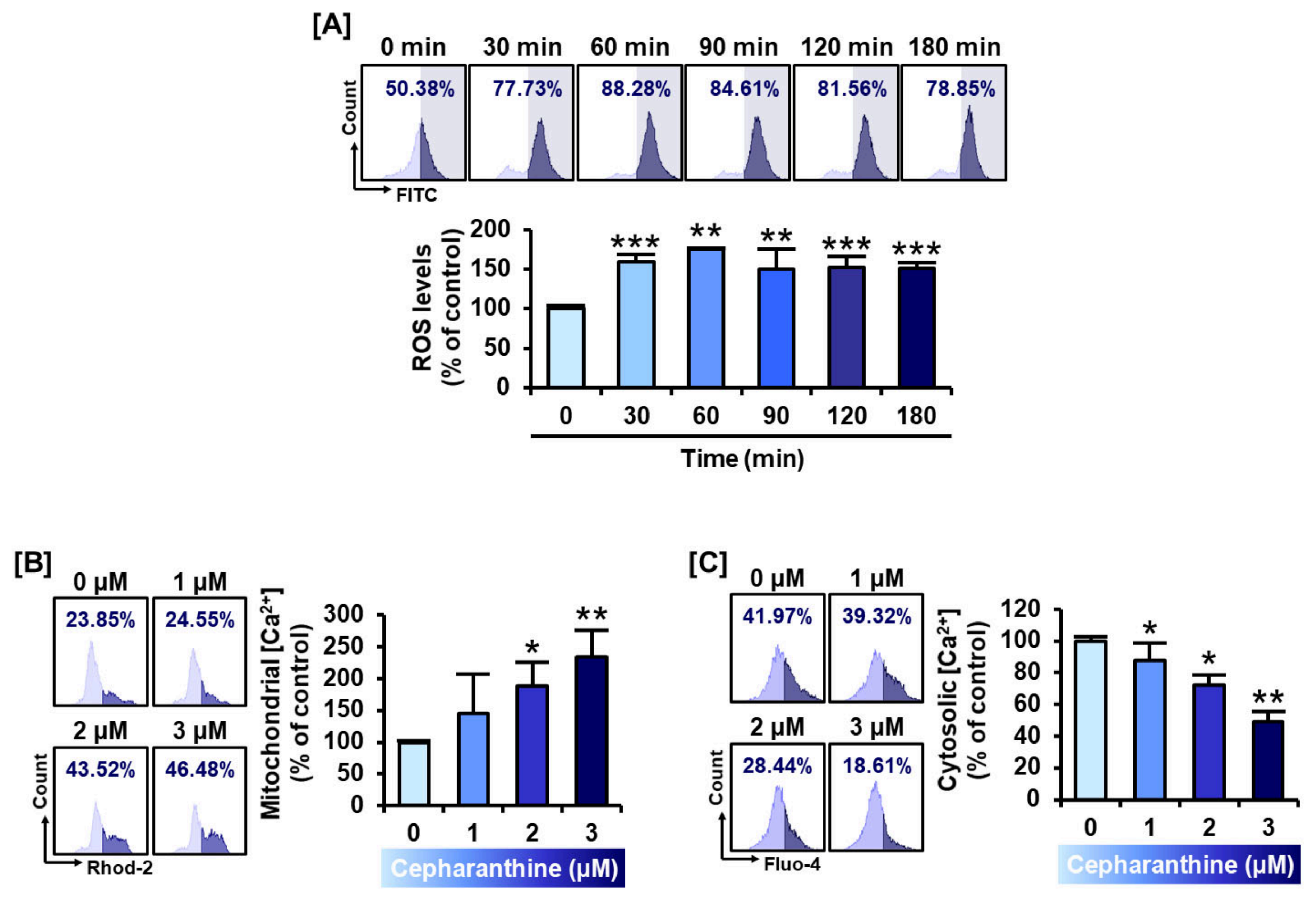
Cepharanthine increases ROS and redistributes intracellular Ca^2+^ in ihOESCs. (**A**) The relative ROS levels in ihOESCs were identified after 3 μM cepharanthine exposure for 0, 30, 60, 90, 120, and 180 min. (**B**) The mitochondrial Ca^2+^ levels and (**C**) cytosolic Ca^2+^ levels were identified. Three independent biological experiments were conducted. Statistics were performed with R programs using one-way ANOVA and Tukey’s test, comparing all pairs of group averages. The asterisks (*) indicated statistical significance: * *p* < 0.05 and ** *p* < 0.01, *** *p* < 0.001.

To identify how cepharanthine induces a response by regulating cell signaling in ihOESCs, we measured ROS production and intracellular Ca^2+^ distribution. To identify the ROS production levels after cepharanthine exposure, oxidant sensitive DCF fluorescence was measured at 0 min and after 30, 60, 90, 120, and 180 min following treatment with 3 μM cepharanthine. Relative DCF fluorescence was significantly increased at each time point (Figure 6A). This indicates increased ROS signals in ihOESCs.

In addition, Ca^2+^ functions as a signaling messenger that regulates various cellular processes. Therefore, we investigated the distribution of Ca^2+^ in the mitochondria and cytoplasm after 48 h of cepharanthine exposure. Mitochondrial Ca^2+^ levels increased by 188.96% (*F*_1,4_ = 19.29, *p* = 0.0118) and 234.26% (*F*_1,4_ = 29.95, *p* = 0.0054) after treatment with 2 μM and 3 μM cepharanthine, respectively (Figure 6B). Furthermore, cytosolic Ca^2+^ levels were significantly reduced after cepharanthine treatment (Figure 6C). These results suggest that cepharanthine induces oxidative stress and mitochondrial Ca^2+^ overload in ihOESCs. Moreover, these intracellular signaling changes may lead to mitochondrial dysfunction and the upregulation of autophagy and apoptotic signals.

The gene expression levels of inflammatory mediator-related genes in ihOESCs presented non-uniform transcriptional changes after cepharanthine treatment (Supplementary Figure S1). Among the transcripts examined, *IL1B* and *IL12A* showed increasing trends (*p* < 0.01), whereas *IL18, IL18R1*, and *IL21* presented significant downregulation (*p* < 0.01). Together, these results indicate that cepharanthine is associated not only with intracellular stress responses but also with gene-specific changes in inflammatory mediator-related gene expressions in ihOESCs.

## 3. Discussion

**Figure 7:**
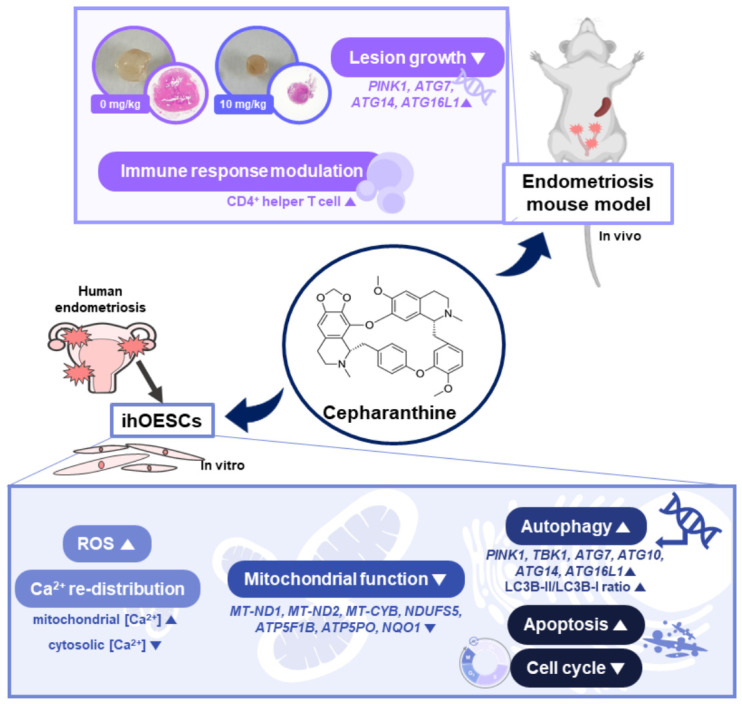
The illustration of cepharanthine-associated responses observed in in vivo and in vitro experiments. Cepharanthine reduced calculated lesion volume and altered autophagy-related gene expression in endometriosis mouse models. In addition, cepharanthine has the potential to modulate immune responses by regulating helper T cell populations. At cellular levels, cepharanthine induced ROS accumulation and Ca^2+^ redistribution, including mitochondrial Ca^2+^ overload, in ihOESCs. These cellular responses were accompanied by mitochondrial dysfunction and autophagy-related signals. The cells also showed an increased apoptotic population and altered cell cycle distribution after cepharanthine treatment. These effects of cepharanthine may contribute to lesion growth inhibition and ameliorate symptoms in patients with endometriosis.

This study investigated the effects and potential mechanisms of cepharanthine in endometriosis using a surgically induced endometriosis mouse model and ihOESCs. Furthermore, using T-HESCs as a non-endometriotic stromal cell comparator, we found that cepharanthine did not induce significant changes in viability or apoptotic cell populations in normal endometrial stromal cells at our experimental concentrations. However, because ihOESCs and T-HESCs are immortalized cell lines, they do not fully reflect the cellular heterogeneity, hormonal environment, or interpatient variability of endometriosis in women. Cepharanthine was associated with a reduction in calculated lesion volume and altered expression of autophagy-related genes in lesion tissues. Cepharanthine was also associated with changes in spleen weight and splenic CD4+ T-cell population in the mouse model. In addition, oxidative stress and Ca^2+^ redistribution were identified in ihOESCs. These findings suggest that cepharanthine suppresses endometriosis-associated lesion volume in the mouse model and alters multiple cellular responses, including mitochondrial dysfunction, cell-cycle distribution, and apoptosis in ihOESCs; these changes may activate cell death signals such as autophagy-related signaling and apoptosis. A schematic representation of these results is shown in Figure 7.

Given that oral administration, a noninvasive method, is commonly used to treat endometriosis, we evaluated the effects of cepharanthine in alleviating endometriosis symptoms [18,19]. The 10 mg/kg dose was selected based on previous studies involving oral administration [20,21]. In this study, plasma and lesion-tissue cepharanthine concentrations were not directly measured; therefore, it is difficult to establish an equivalence between the in vivo concentration of 10 mg/kg and the 3 μM in vitro experimental concentration. A rodent pharmacokinetics study reported that oral administration of cepharanthine at 10 mg/kg has a low maximum plasma concentration (C_max_) and large apparent volume of distribution (Vz) [22]. This suggests extensive tissue distribution despite limited oral bioavailability. Accordingly, the in vitro experimental concentration of 3 μM was selected near the ihOESC IC50 to identify cellular responses, rather than to replicate a measured plasma C_max_.

The administration of 10 mg/kg cepharanthine significantly reduced the calculated lesion volume, whereas wet lesion weight was not significantly altered. Previous studies of surgically induced murine endometriosis have shown that lesions can display cyst-like structural features, which may contribute to discordance between volume and weight-based assessments [16,23]. In the present study, this discrepancy may be related to differences in lesion structure, including cavity size, fluid content, tissue density, or cellularity, which may differentially affect both measurements. The 1 mg readability of the scale may have further reduced the sensitivity of wet weight measurements to these small lesions. Therefore, the decrease in lesion volume should be interpreted as evidence of altered lesion size or structure rather than a confirmed decrease in total lesion mass. Specifically, iron staining in the 10 mg/kg cepharanthine-treated group indicated increased iron accumulation within the tissues. However, this staining was localized and not quantified. Therefore, iron staining alone does not provide direct mechanistic evidence. Nevertheless, local iron-positive signals may be associated with changes in the lesion microenvironment. These changes may be associated with immune-cell activity, oxidative stress, and autophagy-related signals [24,25]. Notably, cepharanthine has been reported to possess iron-chelating activity in other experimental contexts [26,27]. Therefore, the localized Prussian blue signal observed in this study should not be interpreted as evidence of increased total tissue iron, and quantitative iron analyses will be required to clarify its biological meaning.

Additionally, mRNA expression analysis of dissected lesions from the mouse models demonstrated a significant upregulation of autophagy-related genes, including *Pink1, Atg7, Atg14*, and *Atg16l1*. These genes are involved in regulating autophagy [28,29]. Because these gene expressions were assessed at the mRNA levels in heterogeneous lesion tissue, the results indicate transcription changes; protein-level and functional validation are required to determine which corresponding pathways are activated by cepharanthine.

To further investigate the potential mechanism of cepharanthine, we conducted experiments using ihOESCs as an in vitro model. Cepharanthine treatment (0–50 μM) reduced cell viability in a dose-dependent manner, with an IC50 of 3.33 μM. Based on this, subsequent in vitro experiments were conducted at 3 μM cepharanthine, the optimal concentration. In parallel, T-HESCs used as a non-endometriotic stromal comparator did not show significant changes in viability or apoptotic cell populations under the same working conditions. These findings support a differential response between endometriotic and non-endometriotic immortalized stromal cells under our in vitro conditions. Previous studies have reported that cepharanthine induces apoptosis and cell cycle arrest in various diseases, including myeloma, breast cancer, and hepatocellular carcinoma [9,10,30]. Consistent with these findings, cepharanthine treatment in ihOESCs induced apoptosis and altered cell-cycle distribution with decreased S and G2/M phases and an increased Sub G0 phase. Endometriosis lesions are characterized by increased cell proliferation and reduced apoptosis due to dysregulations of the cell cycle and apoptotic signaling [31]. The concordance between MTT and trypan blue assay results supports the conclusion that cepharanthine decreases the number of viable cells rather than causing MTT-specific metabolic effects. These responses may contribute to the lesion-related effects of cepharanthine. Annexin V/PI analysis supports an increase in the apoptotic population after cepharanthine exposure, although the downstream execution pathway was not identified.

Previous studies have also reported the autophagy-related effects of cepharanthine in breast cancer and lung adenocarcinoma cells [10,32]. In this study, AO staining was used to evaluate acidic vesicle-associated signals in ihOESCs after cepharanthine treatment. AO selectively accumulates in acidic organelles, such as lysosomes and autophagosomes, emitting red fluorescence in acidic compartments [33]. Consequently, cepharanthine treatment significantly increased the proportion of red-AO-positive cells in ihOESCs, indicating increased acidic vesicle-associated signals. In addition, cepharanthine upregulated the expression of autophagy-related factors, including *PINK1, TBK1, ATG7, ATG10, ATG14*, and *ATG16L1*, and increased the protein levels of p62/SQSTM1 and LC3B-II [29,34,35]. However, AO staining does not distinguish increased autophagosome formation from lysosomal dysfunction. The accumulation of LC3B-II and p62/SQSTM1 is associated with impaired autophagic degradation rather than simple induction of autophagy [36]. Because a complete flux analysis was not performed, these findings support steady-state accumulation of autophagy-related signals but do not establish increased autophagic flux [37,38].

Among these factors, *PINK1* and *TBK1* are associated with mitophagy, a specialized type of autophagy that eliminates damaged mitochondria [29,39]. Therefore, we investigated the MMP and related gene expression following cepharanthine treatment. Cepharanthine suppressed the expression of mitochondrial ETC components, ATP synthase subunits, and redox balance regulators, and induced mitochondrial depolarization in ihOESCs [40,41]. In addition, cepharanthine treatment elevated ROS levels and mitochondrial Ca^2+^ concentrations, while decreasing cytosolic Ca^2+^ levels. The onset and early kinetics of the ROS response within the first 30 min were not determined because 30 min was the earliest post-treatment time point in this study. These alterations are consistent with mitochondrial and oxidative stress and may trigger autophagy-related signals [42,43]. Notably, cepharanthine has been reported to suppress the generation of reactive oxygen species and exert antioxidant activity in immune cell settings [44,45]. However, in tumor cells, cepharanthine has been reported to induce ROS production accompanied by mitochondrial dysfunction and cell death [7]. Accordingly, ROS responses to cepharanthine may vary across cell types, and we suggest that, under our experimental conditions, cepharanthine in endometriosis cells may activate a cellular stress response through ROS accumulation.

Finally, dysregulated immune responses are critical factors in the progression and symptom severity of endometriosis [46]. In this study, cepharanthine treatment significantly reduced spleen weight in endometriosis mouse models. In environments characterized by ectopic cell growth, such as tumors, spleen weight can be inversely correlated with CD4+ T cell populations, which play a tumor-suppressing role [47]. Alterations in CD4+ T cells have been widely studied in endometriosis [48]. Although the exact role of CD4+ T cells remains controversial, studies have reported lower CD4+ T cell populations in endometriosis lesions compared to normal uterine endometrial tissue [49]. Previous studies have reported that cepharanthine suppresses pro-inflammatory cytokine responses in other experimental systems [20,50]. Exploratory analyses of inflammatory mediator-related transcripts in ihOESCs did not show a uniform anti-inflammatory pattern; rather, cepharanthine was associated with gene-specific changes in these transcripts. This differs from previous reports obtained in immune- or inflammation-driven models, suggesting that inflammatory mediator responses to cepharanthine may vary with cellular context [7,20,50]. Therefore, immune-related interpretation in the present study is limited to spleen weight and the proportion of splenic CD4+ T cells.

This study highlights the potential of cepharanthine as a candidate agent for endometriosis in the in vivo and in vitro models. Under our in vitro conditions, cepharanthine did not induce significant changes in viability or apoptosis in T-HESCs, supporting a differential response between endometriotic and normal endometrial stromal cells. However, direct evaluation in eutopic uterine tissue was not included in the present study. Cepharanthine was associated with mitochondrial dysfunction, ROS signaling, and Ca^2+^ redistribution in ihOESCs, with autophagy-related protein changes, altered cell-cycle distribution, and apoptosis in endometriosis cells. Additionally, it was associated with modest changes in splenic immune cell populations, suggesting that cepharanthine may affect multiple pathological processes in experimental models of endometriosis. While these findings underscore the potential of cepharanthine, further studies are essential to evaluate its long-term safety and efficacy.

In summary, cepharanthine treatment reduced calculated lesion volume in the mouse models. This effect is achieved through mechanisms that impair mitochondrial function, primarily by regulating oxidative stress and Ca^2+^ distribution in ectopic endometriosis cells. Dysfunctional mitochondria may contribute to abnormalities in cell-cycle distribution and to autophagy-induced apoptosis. Furthermore, cepharanthine may modulate spleen weight and splenic CD4+ T-cell population; however, the functional significance of these findings remains to be further investigated. These findings highlight that cepharanthine is a promising candidate for the treatment of endometriosis and provide valuable insights into its underlying mechanism of action.

Limitations of this study include the lack of direct lesion-level immune-cell infiltration analyses (e.g., macrophage markers) and the absence of cytokine profiling, which restricts the interpretation of immune modulation to splenic readouts. Finally, because systemic exposure to cepharanthine was not quantified after oral dosing, direct comparison between the in vivo dose (10 mg/kg) and the in vitro working concentration (3 μM) remains limited in the present study. Therefore, further studies should investigate lesion-level immune profiling, cytokine measurements, and pharmacokinetic analyses to strengthen mechanistic interpretation.

## 4. Materials and Methods

### 4.1. Reagents

Cepharanthine (purity: ≥99.91%) was purchased from MedChemExpress (Cat no. HY-N6972). An amount of 0.5% carboxymethylcellulose (CMC) and dimethyl sulfoxide (DMSO) were used as solvents for cepharanthine in mouse oral administration and cell treatment, respectively. Table 1 provides information on reagents and antibodies used in this paper.

### 4.2. Animal Models

We purchased female C57BL/6J mice (7 weeks old) from DBL (Korea). The purchased mice were confirmed to be negative for disease monitoring tests for viruses, bacteria, mycoplasma, fungi, and parasites. All studies were conducted in compliance with ARRIVE guidelines and fulfilled the requirements of the EU Directive 2010/63/EU for scientific animal studies. The experimental protocols were approved by the Institutional Animal Care and Use Committee (IACUC) at Gyeongsang National University (GNU-230425-M0083). Before the operation, the mice were given a week to adapt to the animal room. The animal room had a 12 h light/dark cycle, and the temperature (23 ± 2 °C) and humidity (50 ± 10%) remained constant. Next to the animal room, there was a treatment room for surgery and experimentation. The water contained 0.0185% HCl and standard chow was provided to mice.

### 4.3. Endometriosis Induction and Treatment

To synchronize the menstrual cycle, all mice were injected subcutaneously with progesterone (2 mg/kg) daily for 2 days before surgery. All animals were anesthetized by intramuscular injection (IM) with Ketamine (Yuhan Corporation, Seoul, Republic of Korea, Cat no. 8806421050721) and Rompun Inj. (Bayer, Leverkusen, Germany, Cat no. 90196434). We followed Pelch’s procedure to construct an endometriosis mouse model [16]. One uterine horn of the mouse was excised and then divided into three identical pieces of 3 mm and placed on the intestinal artery. More detailed information about this operation was provided in a previous study [51]. The surgery was performed in a treatment room. All mice recovered from anesthesia and were transferred to the animal room. After 4 weeks of surgery, mice (*n* = 8) were randomly assigned to two groups (*n* = 4). The sample size was established based on our previous experiment [52,53]. The average weight of mice in both groups was set equal, and each group received either a vehicle (0.5% CMC) or 10 mg/kg cepharanthine orally, using an oral gavage needle, for 4 weeks daily in the treatment room. None of the animals were excluded from the analysis due to complications or deaths related to the effects of the 0.5% CMC or cepharanthine. The in vivo analysis included only mice in which all three lesions were successfully established. Since oral administration of drugs is the standard treatment (e.g., dienogest) for endometriosis, this method was selected for this experiment to compare therapeutic efficacy. After euthanasia, the length, width, and wet weight of the endometriosis lesion and the spleen were measured. Under blinded conditions, lesion volume was estimated from measured length (L) and width (W) using the formula V = (L × W^2^)/2. This value represents a geometric estimate rather than a direct 3D volumetric measurement. Each lesion was measured once. For each mouse, three lesions were quantified, and the mean lesion volume per mouse was used as one biological replicate for statistical analysis. The establishment of the mouse model and the drug administration schedule are presented in Figure 1. Mice were euthanized humanely through CO_2_-induced asphyxiation. After asphyxiation, endometriosis lesions and spleen were collected and stored at −80 °C or in 4% paraformaldehyde (PAF) for subsequent analysis.

### 4.4. Gene Expression Analysis

Total RNA from mouse tissues stored at −80 °C or ihOESCs was extracted by RiboExTM. The extracted RNA was synthesized into complementary DNA (cDNA) using random primers, oligo dT, and AccuPower RT premix, and was used for gene expression analysis by qRT-PCR. qRT-PCR was performed on an AriaMx Real-Time PCR System (Agilent Technologies, Santa Clara, CA, USA) using a SYBR Green based detection chemistry (Sigma-Aldrich) and EasyTaq^®^ DNA polymerase with the corresponding reaction buffer (TransGen Biotech). The total reaction volume was 20 μL, and 120 ng of cDNA template was used per reaction well. Primers were prepared from a 100 μM stock by 10-fold dilution, and 0.5 μL each of forward and reverse primers was added per reaction (final concentration: 0.25 μM each). Amplification was conducted for 40 cycles with the following program: 95 °C for 30 s, 60 °C for 30 s, and 72 °C for 30 s. Melt-curve analysis was performed after amplification to verify product specificity. All primer sequences and amplicon sizes are provided in Table 2. Primer sequences were adopted from previous studies [54,55,56]. Quantification cycle (Cq) values were used for analysis. Target-gene expression was normalized using reference genes, and relative expression was calculated using the 2-ΔΔCq method. Samples with low RNA quality were excluded from statistical analysis, and mRNA expression in vivo was analyzed with *n* = 3. For in vitro RT-qPCR analysis, each sample was measured in technical triplicate within each biological replicate. The in vitro experiment was biologically repeated three times.

### 4.5. Histological Analysis

Collected and stored in 4% PAF, tissues were dehydrated and cleared with xylene and a series of ethanol concentrations. After clearing, tissues were embedded in paraffin and cut to 5 μm for preparing slide glass samples. The histological structure was identified using Mayer’s hematoxylin and eosin Y alcoholic (H&E) staining. In addition, the iron accumulation in tissues was identified by the Prussian blue iron stain kit. The paraffin on each slide of glass was removed with xylene, then washed through low-concentration ethanol in high-concentration ethanol to hydrate the tissue. H&E or iron staining was performed on hydrated tissue, and then dehydration was performed using high-concentration ethanol followed by low-concentration ethanol. Thereafter, the stained tissue slide was fixed with mounting media and a cover glass.

### 4.6. Splenic T Cell Analysis

The dissected spleen was immediately mechanically dissociated (pressed and minced) in a 70 μm cell strainer and resuspended in RPMI-1640 medium with 5% FBS. The RBC lysis buffer was added to remove red blood cells and disrupt spleen tissues in the media. After removing red blood cells, the remaining cells were incubated in 2.4G2 (CD16/32 antibody) solution to block Fc receptors. After blocking, fluorescent labeled antibodies (CD45-PE, CD3-FITC, and CD4-PC5.5) were added to cells to mark lymphocytes, T cells, and helper T cell-specific antigen. A CytoFlex flow cytometer measured the fluorescence of each antibody and gated the total T cell population and helper T cell population. Samples with fewer than 30,000 cells analyzed through the flow cytometer were excluded from statistical analysis, and the analysis proceeded to *n* = 3.

### 4.7. Cell Culture

Immortalized human endometriotic stromal cell lines (ihOESCs) were established previously from an ovarian endometriotic lesion and characterized by Son et al. [17]. ihOESCs were kindly provided by Professor Gwonhwa Song (Korea University, Seoul, Republic of Korea). Cells were grown to 80% confluency in a 37 °C, 5% CO_2_ humidified incubator. The growth medium composition is as follows: DMEM/F-12 media supplemented with 10% FBS, 2 mM of L-glutamine, 1 ng/mL of recombinant human FGF2, and 1% penicillin streptomycin. In addition, to identify the effects of cepharanthine on normal endometrial cells, hTERT-immortalized human endometrial stromal cell lines (T-HESCs) were purchased from the American Type Culture Collection (ATCC, Manassas, VA, USA). T-HESCs were also cultured with DME/F12 1:1 medium without phenol red (Catalog No. D2906, Sigma-Aldrich, Missouri, USA) with 10% charcoal/dextran-coated FBS and 1% CorningTM ITS + Premix universal culture supplement (Catalog No. 354352, Corning, New York, NY, USA). ihOESCs were used as an endometriosis model, and T-HESCs were used as a normal cell control in vitro.

### 4.8. MTT Crystal Production Analysis

Cell viability after treatment with cepharanthine was assessed using Cell Proliferation Kit I. ihOESCs and T-HESCs were seeded on 96-well plates and treated with various concentrations of cepharanthine (0, 1, 2, 5, 8, 10, 20, and 50 μM) for 48 h. The MTT labeling reagents (Roche, Cat no. 11465007001) were added to treated cells for 4 h. After 4 h, the violet crystal formazan was solubilized with the solubilization solution for 18 h. The absorbance was measured, and the IC50 value was calculated using R. The ‘drm’ function in the ‘drc’ package was used to apply the Hill model on raw data. This experiment was performed biologically in triplicate.

### 4.9. Trypan Blue Exclusion Analysis

To identify the viable cell number independently of mitochondrial activity, ihOESCs and T-HESCs were seeded in the 6-well plates and treated with cepharanthine (0, 1, 2, and 3 μM) for 48 h. Cells were harvested and mixed with trypan blue solution (1:1). All cells and viable (dye-excluding) cells were counted using a hemocytometer. The results were presented as % of the control (control set to 100%). This experiment was performed biologically in triplicate.

### 4.10. Evaluation of the Apoptotic Cell Death

ihOESCs were seeded on 6-well plates to 50% confluency and treated with cepharanthine (0, 1, 2, and 3 μM) for 48 h. As a non-endometriotic comparator, T-HESCs were also treated with the same concentration of cepharanthine for 48 h. Cells were harvested and resuspended in 1× annexin binding buffer. The cells were stained with Annexin V dye (BD Bioscience, Cat no. 556419) and propidium iodide (PI, Sigma-Aldrich, Cat no. P4864) for 15 min. The fluorescent signals from stained cells were acquired using a CytoFlex flow cytometer (Beckman Coulter, Brea, CA, USA). The interference between Annexin V and PI signals was compensated. This experiment was performed biologically in triplicate.

### 4.11. Evaluation of the Cell Cycle Distribution

ihOESCs were prepared as described in Section 4.10. To identify the cell cycle distribution of ihOESCs, the cells were harvested and fixed in 70% ethanol for 24 h. Fixed cells were incubated with RNase A and PI for 30 min. The fluorescence signal of PI reflects the amount of DNA, and the cell cycle phase distribution can be identified based on signal intensity. The fluorescent signals were identified with a CytoFlex flow cytometer. This experiment was performed biologically in triplicate.

### 4.12. Evaluation of Acidic Vesicle-Associated Signals

Cells were prepared as described in Section 4.10. Cepharanthine-treated cells were harvested with 0.05% Trypsin-EDTA and washed with PBS. The cells were stained with acridine orange staining solution for 20 min. The stained cells were washed with PBS once and detected by a CytoFlex Flow cytometer. This experiment was biologically triplicated.

### 4.13. Evaluation of Protein Expression Levels

Cells were seeded in 60 mm plates and treated with vehicle or 3 μM cepharanthine for 48 h. After treatment, cells were lysed in a lysate buffer containing a protease inhibitor cocktail. Total protein was extracted from lysed cells. Protein concentrations were quantified using the Bradford assay with albumin standard. Protein loading samples were incubated at 95 °C for 5 min, and 20 μg of total protein was loaded per lane. Proteins separated by size on 10% SDS-PAGE gels were transferred to a nitrocellulose membrane and incubated with a primary antibody against p62/sQSTM1, LC3B, and α-tubulin (1:1000 dilution) overnight at 4 °C on a shaker. Secondary antibodies for signal detection were then further processed and measured with the VILBER chemiluminescence imaging system. The signal for the target protein was normalized to that of α-Tubulin, a housekeeping protein. The values were presented as % of the control (control set to 100%). This experiment was performed biologically in triplicate.

### 4.14. Evaluation of the Mitochondrial Membrane Potential (MMP)

ihOESCs were prepared as described in Section 4.10. Cepharanthine-treated cells were resuspended in staining solution containing JC-1 dye for 20 min. JC-1 aggregates in healthy mitochondria to emit red fluorescence and is present as a monomer of green fluorescence when the MMP is depolarized. The fluorescent signals of JC-1 dye were identified with a CytoFlex flow cytometer. This experiment was performed biologically in triplicate.

### 4.15. Evaluation of the Intracellular Reactive Oxygen Species (ROS) Level

ihOESCs were prepared in a 6-well plate at 80% confluency. The cells were treated with 3 μM cepharanthine for 0, 30, 60, 90, 120, and 150 min. After treatment, 0.05% Trypsin-EDTA was used to harvest cepharanthine-treated cells. The cells were stained with 2′,7′-dichlorodihydrofluorescein diacetate (DCFH-DA; Sigma-Aldrich, Cat no. 35848) for 30 min, and the fluorescent signals generated from 2′,7′-dichlorofluorescein (DCF) were detected using the CytoFlex flow cytometer. This experiment was performed biologically in triplicate.

### 4.16. Measurement of Intracellular Ca^2+^ Distribution

ihOESCs were seeded in a 6-well plate at 40% confluency and treated with 0, 1, 2, and 3 μM cepharanthine for 48 h. The cells were collected using 0.05% Trypsin-EDTA and prepared for fluorescent dye staining. To determine mitochondrial Ca^2+^ levels, the Rhod-2 AM dye (Invitrogen, Cat no. R1244) was diluted in HBSS without Ca^2+^ and Mg^2+^. The cells were incubated with Rhod-2 AM staining solution for 20 min. To investigate the cytosolic Ca^2+^ levels, Fluo-4 AM dye (Invitrogen, Cat no. F14201) was utilized. The Fluo-4 AM dye was diluted in serum-free media and DMSO, and cells were stained in the prepared staining solution. The fluorescent signals of stained cells by Rhod-2 AM or Fluo-4 AM were analyzed with a CytoFlex flow cytometer. This experiment was biologically triplicated.

### 4.17. Statistical Analysis

The statistical significance of quantitative data was analyzed using R. For two-group comparisons, a two-tailed unpaired Student’s *t*-test was applied. For experiments with three or more groups, statistical significance was confirmed by ANOVA (one-way analysis of variance) and Tukey’s test. The functions “aov()” and “TukeyHSD()” from the “stats” library in the R program were applied. An asterisk (*) in the figure indicates that the *p*-value was less than 0.05, implying statistical significance. The F value was expressed with the degrees of freedom (df). Fab (a: df between groups, b: df within groups).

## Figures and Tables

**Table 1:** The detailed information on reagents and antibodies used in this study.

Names	Catalog No.	Sources
Cepharanthine	HY-N6972	MedChemExpress (Monmouth Junction, NJ, USA)
Carboxymethylcellulose sodium salt	C5678	Sigma-Aldrich (St. Louis, MO, USA)
Dimethyl sulfoxide	D8418	Sigma-Aldrich (St. Louis, MO, USA)
RiboExTM	301-001	GeneAll^®^ (Seoul, Republic of Korea)
Random primers	48190011	Invitrogen (Carlsbad, CA, USA)
AccuPower RT premix	K-2055B	Bioneer (Daejeon, Republic of Korea)
EasyTaq^®^ DNA polymerase	AP111-01	TransGen Biotech (Beijing, China)
Mayer’s hematoxylin	CM3953	Cancer Diagnostics (Durham, NC, USA)
Eosin Y alcoholic	EM500G	Cancer Diagnostics (Durham, NC, USA)
Prussian blue iron stain kit.	AB150674	Abcam (Cambridge, UK)
RPMI-1640 medium	SH30027.01	HyClone (Logan, UT, USA)
Fetal bovine serum (FBS)	SH30919.03	HyClone (Logan, UT, USA)
RBC lysis buffer	555899	BD Biosciences (Franklin Lakes, NJ, USA)
Rat anti-mouse CD16/CD32	553141	BD Biosciences (Franklin Lakes, NJ, USA)
PE anti-mouse CD45 antibody	103105	BioLegend (San Diego, CA, USA)
FITC anti-mouse CD3ε antibody	100306	BioLegend (San Diego, CA, USA)
CD4 monoclonal antibody, PC5.5	45-0042-82	Invitrogen (Carlsbad, CA, USA)
DMEM/F-12 medium	SH30023.01	HyClone (Logan, UT, USA)
L-glutamine	25030081	Gibco (Grand Island, NY, USA)
Recombinant human FGF_2_	ab9596	Gibco (Grand Island, NY, USA)
Penicillin streptomycin	SV30010	HyClone (Logan, UT, USA)
Cell proliferation kit I	11465007001	Roche (Basel, Switzerland)
FITC annexin V dye	556419	BD Biosciences (Franklin Lakes, NJ, USA)
Propidium iodide (PI)	P4864	Sigma-Aldrich (St. Louis, MO, USA)
Acridine orange	A3568	Invitrogen (Carlsbad, CA, USA)
Halt^TM^ Protease inhibitor cocktail	87785	Thermo (Waltham, MA, USA)
LC3B (D11) antibody	3868S	Cell Signaling Technology (Danvers, MA, USA)
SQSTM1/P62 antibody	88588S	Cell Signaling Technology (Danvers, MA, USA)
α-Tubulin antibody (DM1A)	sc-32293	Santa Cruz (Santa Cruz, CA, USA)
Affinity purified antibody peroxidase labeled goat anti-rabbit	474-1506	KPL (Gaithersburg, MD, USA)
Affinity purified antibody peroxidase labeled goat anti-mouse	474-1806	KPL (Gaithersburg, MD, USA)
JC-1 dye	T4069	Sigma-Aldrich (St. Louis, MO, USA)
DCFH-DA	35848	Sigma-Aldrich (St. Louis, MO, USA)
Rhod-2 AM	R1244	Invitrogen (Carlsbad, CA, USA)
HBSS without Ca^2+^, Mg^2+^ medium	14175095	Gibco (Grand Island, NY, USA)
Fluo-4 AM	F14201	Invitrogen (Carlsbad, CA, USA)

**Table 2:** The primer sets of target genes used in quantitative real time PCR.

Species	Gene (Size)	Primer Sequence (5′→3′)	
*Mus musculus*	*Pink1* (77 bp)	F R	GAACTACCCCTGTACCCTGC GCAAGGTCATCATGGTAGCC
	*Tbk1* (85 bp)	F R	GCCGGGAACAACTCAATACC TCCCCATCCAGATCATAGCG
	*Atg7* (51 bp)	F R	TCAGGTCCAGAACAGAAGCC TTCCTGAGACCTGCTAAGCC
	*Atg10* (144 bp)	F R	CAGACATTCACAGCAGATAGGC TCTGTAGGGAGACATGTGAGC
	*Atg14* (137 bp)	F R	CATGGAGCATAACAACCCCG GGTTTTCGCCACAGAACTCG
	*Atg16l1* (56 bp)	F R	GTGGACAAAGGAAGCAGAGC CTAAGGGTGCAGCATCAAGG
*Homo sapiens*	*PINK1* (146 bp)	F R	AGTTTGGCTGTGACCTTTGC CCAGGCCTCATGTAATTTCC
	*TBK1* (112 bp)	F R	TTAGGCCAAGGAGCTACTGC GAACATCCACTGGACGAAGG
	*ATG7* (123 bp)	F R	AGTTGTTTGCTTCCGTGACC TCCCATCCAACTGCTTTAGG
	*ATG10* (116 bp)	F R	TACTTCATCCCTGCAAGACG AACAACTGGCCCTACAATGC
	*ATG14* (94 bp)	F R	ACGATCACAACGGAGACACC TCCACCCAGCTGTAGTAGGC
	*ATG16L1* (145 bp)	F R	CAAGGTGGTGTTCTCTGTGC AGGGAGGGGTCTGTAGTTCG
	*MT-ND1* (80 bp)	F R	TAGCAGAGACCAACCGAACC GGCGTATTCGATGTTGAAGC
	*MT-ND2* (108 bp)	F R	CCGGACAATGAACCATAACC CTGGGACTCAGAAGTGAAAGG
	*MT-CYB* (117 bp)	F R	ACTACAACCCTTCGCTGACG AGAAGAGCGATGGTGAGAGC
	*NDUFS5* (87 bp)	F R	TGAACAGCCCTACAAGATGG GCCCGAGTATAACCGATTCC
	*ATP5F1B* (120 bp)	F R	TCCTGTTGGTCCTGAGACTT ATGAACTCTGGAGCCTCAGC
	*ATP5PO* (137 bp)	F R	ATCCCTATGTGAAGCGTTCC AACGACTCCTTGGGTATTGC
	*NQO1* (138 bp)	F R	GAAATTTGGCCTTTCTGTGG GGAAGCCTGGAAAGATACCC

## Data Availability

Data will be made available on request.

## Supplementary Materials

The following supporting information can be downloaded at: https://www.mdpi.com/article/10.3390/molecules31152722/s1. Figure S1: cepharanthine selectively regulates transcriptional levels of inflammatory mediator-related genes in ihOESCs. [A] Relative mRNA expression levels of inflammatory cytokines and cytokine receptors (IL1B, IL6, IL4R, IL12a, IL13, IL18, IL18R1, IL21, and IL21R) were altered in ihOESCs treated with cepharanthine. The experiments were triplicated biologically. Statistical analyses were performed using the R program using one-way ANOVA and Tukey’s test. The asterisks (*) indicated statistical significance: * *p* < 0.05 and ** *p* < 0.01.

## Abbreviations

The following abbreviations are used in this manuscript:

DSS	dextran sulfate sodium
ROS	reactive oxygen species
ihOESCs	immortalized human ovarian endometriotic stromal cell lines
T-HESCs	hTERT-immortalized human endometrial stromal cells
CMC	carboxymethylcellulose
DMSO	dimethyl sulfoxide
DCFH-DA	2′,7′-dichlorodihydrofluorescein diacetate
ETC	electron transport chain
AO	acridine orange
PINK1	PTEN-induced kinase 1
TBK1	TANK-binding kinase 1
ATG	autophagy-related
LC3B	microtubule-associated protein 1 light chain 3 beta
PI	propidium iodide
MMP	mitochondrial membrane potential
MT-ND1	mitochondrially encoded NADH dehydrogenase 1
MT-ND2	mitochondrially encoded NADH dehydrogenase 2
MT-CYB	mitochondrially encoded cytochrome b
NDUFS5	NADH ubiquinone oxidoreductase subunit S5
ATP5F1B	ATP synthase F1 subunit beta
ATP5PO	ATP synthase peripheral stalk subunit OSCP
NQO1	NAD(P)H quinone dehydrogenase 1
