# Supplementary material for: Cepharanthine Reduces Endometriosis Lesions and Alters Mitochondrial and Autophagy-Related Signals in Endometriotic Stromal Cells
Source: Molecules. 2026 Aug 5;31(15):2722. doi: 10.3390/molecules31152722 (PMC13468403; doi:10.3390/molecules31152722)
Supplement: Supplementary file 1 [file molecules-31-02722-s001.zip › molecules-4428562-supplementary.pdf]

[A]

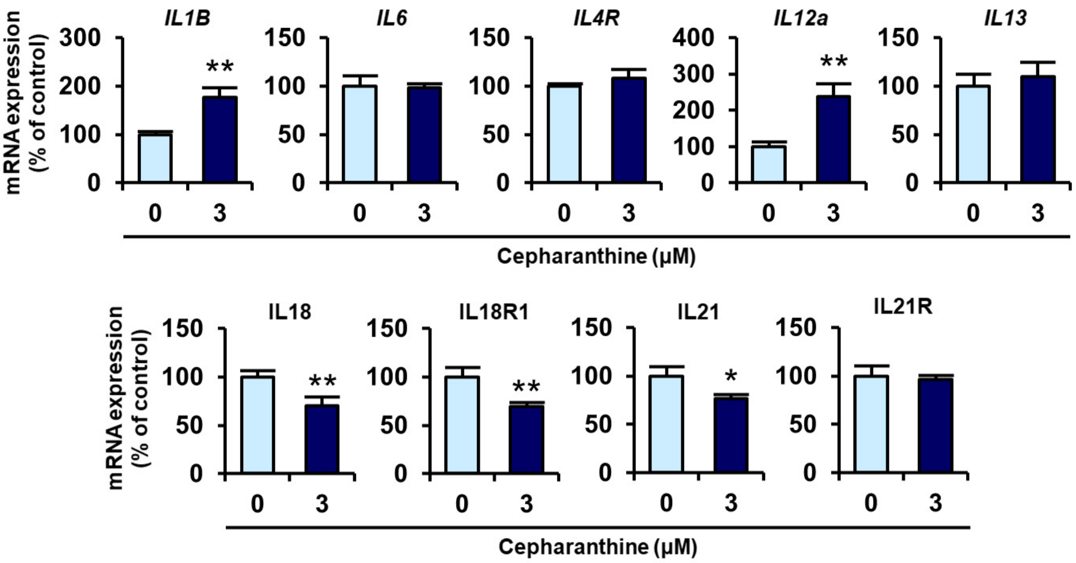

Supplementary Figure S1. *Cepharanthine selectively regulates transcriptional levels of inflammatory mediator-related genes in ihOESCs. [A] Relative mRNA expression levels of inflammatory cytokines and cytokine receptors (IL1B, IL6, IL4R, IL12a, IL13, IL18, IL18R1, IL21, and IL21R) were altered in ihOESCs treated with cepharanthine. The experiments were triplicated biologically. Statistical analyses were performed using the R program using one-way ANOVA and Tukey's test. The asterisks (*) indicated statistical significance: * $p < 0.05$ and ** $p < 0.01$.*
